# CovalentInDB 2.0: an updated comprehensive database for structure-based and ligand-based covalent inhibitor design and screening

**DOI:** 10.1093/nar/gkae946

**Published:** 2024-10-23

**Authors:** Hongyan Du, Xujun Zhang, Zhenxing Wu, Odin Zhang, Shukai Gu, Mingyang Wang, Feng Zhu, Dan Li, Tingjun Hou, Peichen Pan

**Affiliations:** College of Pharmaceutical Sciences, Zhejiang University, Hangzhou 310058, Zhejiang, China; College of Pharmaceutical Sciences, Zhejiang University, Hangzhou 310058, Zhejiang, China; College of Pharmaceutical Sciences, Zhejiang University, Hangzhou 310058, Zhejiang, China; College of Pharmaceutical Sciences, Zhejiang University, Hangzhou 310058, Zhejiang, China; College of Pharmaceutical Sciences, Zhejiang University, Hangzhou 310058, Zhejiang, China; College of Pharmaceutical Sciences, Zhejiang University, Hangzhou 310058, Zhejiang, China; College of Pharmaceutical Sciences, Zhejiang University, Hangzhou 310058, Zhejiang, China; College of Pharmaceutical Sciences, Zhejiang University, Hangzhou 310058, Zhejiang, China; College of Pharmaceutical Sciences, Zhejiang University, Hangzhou 310058, Zhejiang, China; College of Pharmaceutical Sciences, Zhejiang University, Hangzhou 310058, Zhejiang, China

## Abstract

The rational design of targeted covalent inhibitors (TCIs) has emerged as a powerful strategy in drug discovery, known for its ability to achieve strong binding affinity and prolonged target engagement. However, the development of covalent drugs is often challenged by the need to optimize both covalent warhead and non-covalent interactions, alongside the limitations of existing compound libraries. To address these challenges, we present CovalentInDB 2.0, an updated online database designed to support covalent drug discovery. This updated version includes 8303 inhibitors and 368 targets, supplemented by 3445 newly added cocrystal structures, providing detailed analyses of non-covalent interactions. Furthermore, we have employed an AI-based model to profile the ligandability of 144 864 cysteines across the human proteome. CovalentInDB 2.0 also features the largest covalent virtual screening library with 2 030 192 commercially available compounds and a natural product library with 105 901 molecules, crucial for covalent drug screening and discovery. To enhance the utility of these compounds, we performed structural similarity analysis and drug-likeness predictions. Additionally, a new user data upload feature enables efficient data contribution and continuous updates. CovalentInDB 2.0 is freely accessible at http://cadd.zju.edu.cn/cidb/.

## Graphical Abstract

**Graphical Abstract:**
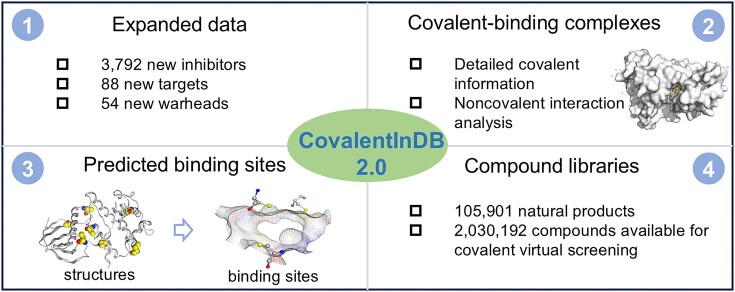

## Introduction

Over the past 30 years, the rational design of targeted covalent inhibitors (TCIs) has garnered significant interest in the pharmaceutical industry (1). TCIs are characterized by two key components: a bond-forming functional group of low reactivity, commonly referred to as the ‘warhead’, and a selective noncovalent fragment for target recognition (2). These structural characteristics confer unique advantages to TCIs, including stronger binding affinity and prolonged target engagement, which can result in distinct pharmacodynamic profiles and exceptional potency (3). Additionally, TCIs have enabled the targeting of traditionally ‘undruggable’ proteins, exemplified by the approval of sotorasib (4,5).

Despite these advantages, the development of covalent drugs remains challenging. Between 2020 and 2024, the FDA approved a total of 127 small molecule chemical drugs, of which only six were covalent drugs. Several factors contribute to this difficulty, including the need to carefully balance the reactivity of the warhead with its proximity to nucleophilic amino acids, and the necessity to finely tune the structure of the non-covalent portion, as inhibitors primarily recognize and bind to the receptor pocket through non-covalent interactions (6,7). Existing databases like CovPDB (8) and CovBinderInPDB (9), while useful in organizing cocrystal structures and covalent binding information, fall short in analyzing the non-covalent interactions within these structures. Furthermore, the limited number of nucleophilic amino acids currently utilized in covalent drug design constrains the exploration of covalent inhibitor binding sites across the proteome (10). Additionally, the small size of existing covalent compound libraries restricts the exploration of chemical space for covalent inhibitors (11). There is a significant need for larger compound libraries to enhance covalent inhibitor design and screening.

The first version of CovalentInDB (12) was developed to provide structural information and experimental data for covalent inhibitors, including annotated warhead, reaction mechanism, and covalent binding site information. It offered convenient functions for data retrieval, browsing, and downloading. Over the past four years, the database has gained widespread attention, with nearly 140 000 visits from 82 countries.

To address the challenges mentioned above and to reduce barriers in the covalent drug discovery process, we have significantly updated CovalentInDB. The expanded dataset now includes 8303 inhibitors (up from 4511) and 368 targets (up from 280). Additionally, we have introduced three new data types to specifically tackle these challenges. First, we collected and organized 3445 cocrystal structures of covalent inhibitors and their targets, systematically analyzing the non-covalent interactions between ligands and receptors. Second, using an AI-based model, we profiled the ligandability of 144 864 cysteines in the proteome, facilitating the discovery of potential covalent binding sites. Third, we constructed a natural product compound library with covalent binding potential, containing 105 901 molecules, and created the largest known covalent virtual screening library with 2 030 192 commercially available compounds. This extensive library fulfills a critical need for large-scale resources in covalent drug screening and discovery. We also conducted structural similarity analysis and drug-likeness predictions on these compounds, enhancing their utility for identifying and developing new covalent drugs. To further enhance the database, we introduced a user data upload feature, enabling the scientific community to contribute and update data more efficiently. These updates significantly enhance the utility of CovalentInDB, making it a more powerful and comprehensive resource for covalent drug discovery.

## Materials and methods

### Update of covalent inhibitor data

Covalent inhibitor data and target information were primarily sourced from scientific literature and several established databases, including ChEMBL (13), DrugBank (14) and UniProt (15), following the methodology employed in the first version of CovalentInDB. We performed a systematic search on PubMed using keywords such as ‘covalent’, ‘covalently’, ‘irreversible’ and ‘irreversibly’. The search results were filtered based on the titles and abstracts to identify research papers specifically related to covalent inhibitors. For each identified covalent inhibitor, we manually extracted detailed covalent binding information, including the warhead, reaction mechanism, binding site, and experimental methods used to validate covalent binding. Additionally, activity data and target information were obtained from ChEMBL and UniProt.

### Covalent inhibitor-target cocrystal structures

Cocrystal structures were sourced from three databases: RCSB PDB (16), CovPDB (8) and CovBinderInPDB (9). Covalent binding was identified by analyzing the ‘LINK’ entries in PDB structure files. We manually reviewed and retained structures containing covalent inhibitors, extracting critical information such as the warhead type, reaction mechanism and pre-reaction structures from the original literature. The UniProt ID of each target and the sequence number of the reaction residue were obtained using SIFTS (17). To support structure-based covalent drug design, we performed a detailed analysis of the interactions between inhibitors and their targets in each cocrystal structure. These interactions included hydrogen bonds, hydrophobic interactions, π–π stacking, π–cation interactions, halogen bonds and salt bridges, which were analyzed using the Open Drug Discovery Toolkit (ODDT) (18). These interactions were visualized using in-house scripts and the 3Dmol.js plugin (19).

### Profiling covalent binding sites with an AI-based model

To address the limited exploration of binding sites in covalent inhibitor development, we employed our previously developed AI model, DeepCoSI, which uses graph deep learning to predict the covalent ligandability of cysteines in protein structures (20). This model has demonstrated state-of-the-art predictive capabilities. We applied DeepCoSI to human protein structures with resolutions higher than 2 Å from the PDB database, ranking the ligandability of cysteines in each structure. AncPhore (21) was used to analyze the pharmacophore characteristics of each potential binding site, with the results visualized using the 3Dmol.js plugin.

### Covalent natural product and virtual screening library

The structural information for natural products was sourced from the COCONUT database (22), which compiles data from 50 open natural product resources, making it one of the largest and well-annotated resources available. Compounds in the virtual screening library were sourced from ZINC20 (23), a database of commercially available compounds for virtual screening. We selected 15 warheads commonly found in covalent inhibitors and conducted substructure analysis on the compounds in these libraries, identifying 105 901 natural products and 2 030 192 compounds with covalent binding potential. ADMET Lab 2.0 was used to predict the drug-likeness of these compounds (24), and Morgan molecular fingerprints were utilized to calculate the Tanimoto structural similarity of each compound with known covalent inhibitors and drugs. These extensive covalent compound libraries, along with the predicted properties, are expected to significantly enhance the discovery of covalent drugs.

## Results

### Data overview

In this updated release of CovalentInDB 2.0, we have significantly expanded the database's content and capabilities compared to the previous version (Table 1). The number of covalent inhibitors has nearly doubled, increasing from 4511 to 8303. The database's scope has also broadened, with the number of unique targets rising from 280 to 368, and the number of approved drugs increasing from 68 to 75. A notable enhancement is the comprehensive cataloging of 111 distinct warhead types, doubling the previous count of 57. In addition to these expansions, we have integrated four new types of data into the database. First, we compiled 3445 cocrystal structures of covalent inhibitors and their target proteins, providing detailed structural insights into protein-ligand interactions. For the first time, we utilized the AI model DeepCoSI (20) to profile 144 864 covalent binding sites from 40 098 high-resolution human protein structures, significantly enhancing the database's utility for identifying potential binding sites. Recognizing the need for a comprehensive compound library for covalent inhibitor discovery, we identified 105 901 natural products with covalent binding potential and created a covalent virtual screening library containing 2 030 192 commercially available compounds, complete with predicted absorption, distribution, metabolism, excretion, and toxicity (ADMET) properties. These expansions substantially broaden the dataset available in CovalentInDB, making it an essential resource for the discovery and development of covalent inhibitors.

**Table 1.** Data statistics of CovalentInDB 1.0 and 2.0

Data category	Version 1.0	Version 2.0
Number of inhibitors	4511	8303
Number of targets	280	368
Number of drugs	68	75
Number of warhead types	57	111
Number of cocrystal structures	–	3445
Number of profiled covalent binding sites	–	144 864
Number of natural products with covalent binding potential	–	105 901
Number of compounds in covalent virtual screening library	–	2 030 192

### Access and display of new features

#### Covalent inhibitor-target cocrystal structures

**Figure 1.**
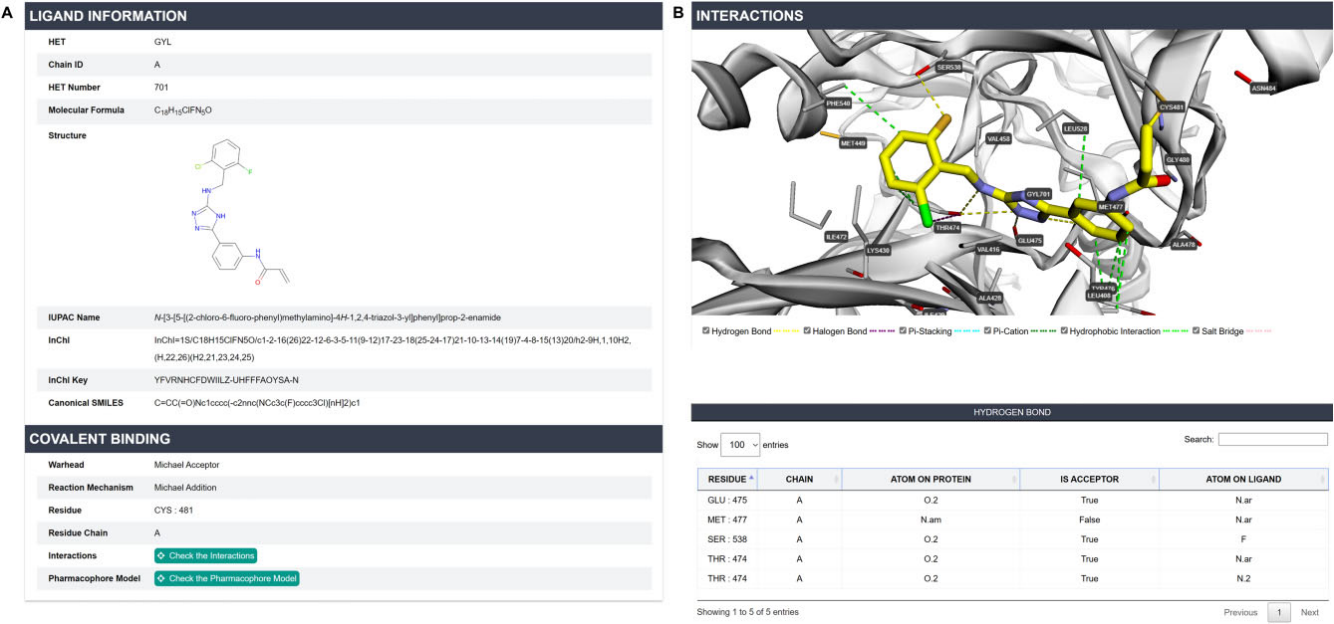
(**A**) Structure of the ligand and covalent binding information within the cocrystal structure of the covalent inhibitor-target complex. (**B**) Non-covalent interactions between the ligand and receptor.

The expanded database now provides users with an extensive collection of cocrystal structures, accessible through an intuitive search interface. Users can search by entering a target's UniProt ID in the homepage search bar, displaying all relevant cocrystal structure data in a tabular format, including target name, covalent binding site, warhead type, and covalent reaction mechanism. Alternatively, users can enter the PDB ID directly to access specific PDB pages. Each PDB entry offers structural information and detailed covalent binding data, such as the covalent inhibitor's serial number, original structure, warhead, reaction mechanism, and binding site (Figure 1A). Additionally, a link is provided to further explore other covalent inhibitors and associated activity data for this target.

To support structure-based covalent inhibitor design, we analyzed the interactions between each covalent ligand and its receptor, focusing on six key intermolecular interactions critical in drug design: hydrogen bonds, hydrophobic interactions, π–π stacking, π–cation interactions, halogen bonds and salt bridges. The 3Dmol.js plug-in (19) is embedded in the webpage, allowing users to visualize these interactions within the binding pocket (Figure 1B). Interactive features enable users to selectively display or hide specific interaction types. Furthermore, a table details the residues and atoms involved in these interactions. We have also developed pharmacophore models based on these interactions, aiding in the design of covalent inhibitors targeting specific structures.

#### Profiled covalent binding sites

**Figure 2.**
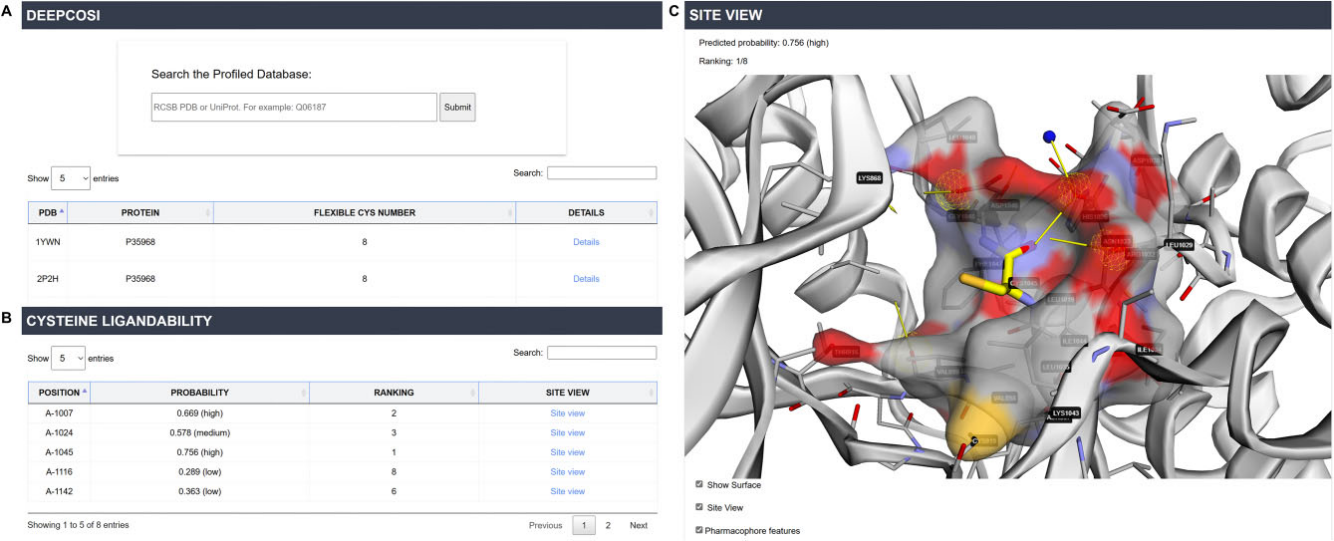
(**A**) Acquisition of profiled cysteine ligandability data. (**B**) Ranking of ligandability for all flexible cysteines in a protein structure (2P2H). (**C**) Display of potential covalent binding sites and surrounding pharmacophore features in ‘Site View’ mode.

Similar to the cocrystal structure data, users can search for profiled binding site data by UniProt ID or PDB ID from the homepage (Figure 2A). For each PDB entry, the AI model DeepCoSI predicts the ligandability of flexible cysteines within the structure (Figure 2B). Users can access detailed site information by clicking the ‘Site View’ button, allowing them to examine pocket characteristics such as shape and depth (Figure 2C). Pharmacophore characteristics of each potential binding site are also analyzed and displayed, providing guidance for designing covalent inhibitors.

#### Natural products and covalent virtual screening library

**Figure 3.**
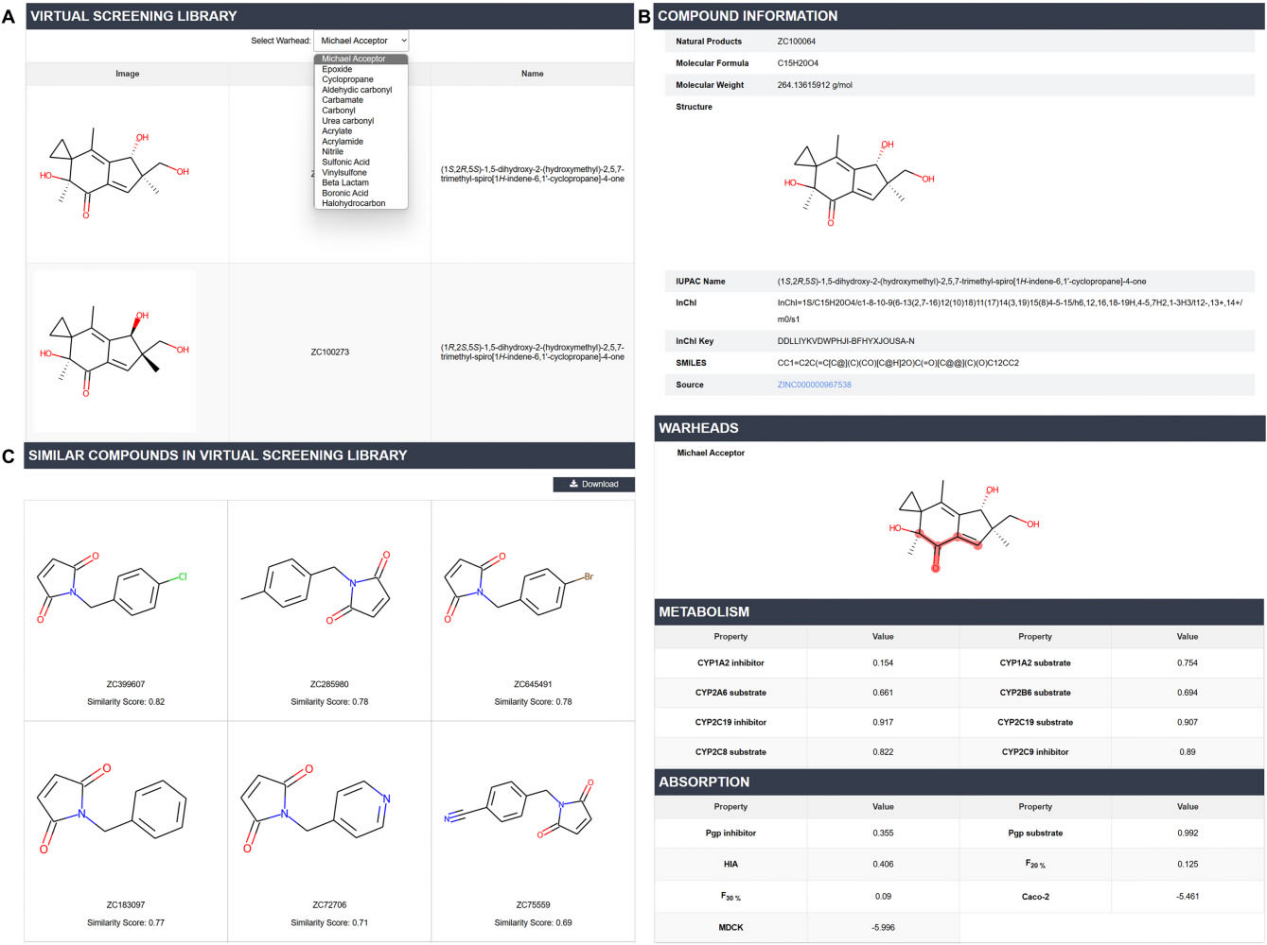
(**A**) Browsing the two libraries by warhead type. (**B**) Structure, warhead, and ADMET properties of natural products or virtual screening compounds. (**C**) Compounds in the covalent virtual screening library with structures similar to the inhibitor CI002722.

Users can explore natural products with covalent binding potential and compounds in the covalent virtual screening library by selecting the ‘browse’ option in the top navigation bar. These two libraries are essential resources for virtual screening of covalent inhibitors, offering significant advantages for identifying promising lead compounds. We are the first to develop such a comprehensive covalent virtual screening library, providing unprecedented convenience to the covalent drug discovery community. Our focus includes 15 commonly used warheads in covalent inhibitors, such as acrylamide, acrylate, halohydrocarbon, nitrile, and vinylsulfone. Users can choose specific warhead categories to view related compounds (Figure 3A), accessing detailed structural and property information via the download function. Each compound page provides comprehensive data, including the InChI, InChI key, IUPAC name and highlights of all covalent warhead substructures (Figure 3B). The predicted ADMET properties, generated using ADMET Lab 2.0 (24), encompass 53 ADMET parameters and 10 physicochemical properties, which are critical for assessing the viability and safety of potential drugs in the drug discovery process. Additionally, we have calculated structural similarities between each compound and known covalent inhibitors, highlighting those with a similarity greater than 0.5. On the covalent inhibitor and covalent drug pages, users can view natural products and compounds in the covalent virtual screening library that have structures similar to the inhibitors (Figure 3C). A convenient download function is also provided to facilitate ligand-based covalent drug discovery, providing researchers with the tools necessary to advance the identification and development of new therapeutic compounds.

#### Data upload feature

To leverage the collective expertise of the scientific community and facilitate timely updates to the database, we have implemented a user data upload feature. Accessible via the ‘Deposit’ option in the top navigation bar, this feature supports two methods of data submission: single molecule mode and multi-molecule mode. In single molecule mode, users can define the chemical structure by drawing it, pasting a SMILES string, or uploading a structure file. Alongside the structural information, users can provide additional details such as target, warhead, reaction mechanism, and covalent binding site. They can also submit five types of activity data: ‘Target Inhibition’ to describe the covalent inhibition effect, ‘Bioactivity’ for cellular or organism-level activity, ‘Selectivity’ indicating the compound's inhibitory capacity on other targets, ‘ADMET’ properties, and ‘Reactivity’ for intrinsic covalent reactivity, often assessed via the compound's interaction with glutathione. The multi-molecule mode allows users to upload data for multiple compounds simultaneously using a tabular format, with an example file provided for guidance. All user-submitted data undergo a thorough manual review to ensure accuracy and quality before being incorporated into the database.

## Conclusion

CovalentInDB 2.0 addresses key challenges in the development of TCIs by significantly expanding its dataset and introducing advanced features. With 8303 inhibitors and 368 targets, the database now offers a broader and more comprehensive resource. The inclusion of 3445 cocrystal structures, complete with detailed non-covalent interaction analyses, enhances our understanding of ligand-receptor dynamics essential for rational drug design. The AI-based profiling of 144 864 cysteines across the human proteome represents a major advancement, overcoming limitations of targeting a narrow range of nucleophilic amino acids and identifying potential covalent binding sites more effectively. Furthermore, the construction of the largest covalent virtual screening library, comprising 2 030 192 commercially available compounds, and a natural product library with 105 901 molecules, provides essential resources for large-scale covalent drug screening and discovery. Structural similarity analyses and drug-likeness predictions enhance the practical utility of these compounds, facilitating both ligand-based and structure-based covalent inhibitor virtual screening. The new user data upload feature fosters community contributions, ensuring the database remains current and dynamic, and accelerates the discovery process by leveraging collective expertise. In summary, CovalentInDB 2.0 significantly enhances its predecessor, providing a comprehensive and versatile resource for covalent drug discovery. By addressing critical challenges and expanding access to high-quality data, CovalentInDB 2.0 is poised to substantially advance the field of covalent drug development.

## Data Availability

CovalentInDB 2.0 is freely accessible at http://cadd.zju.edu.cn/cidb/.
